# Compatible co-administration of BioThrax® vaccine and ciprofloxacin—Results of a randomized open-label drug-vaccine interaction trial

**DOI:** 10.1016/j.jvacx.2024.100431

**Published:** 2024-01-07

**Authors:** David Cassie, Janice Longstreth, Robert Hopkins, Ericka Hunter-Stitt, Bojan Drobic, Melisa Bellani

**Affiliations:** a Emergent BioSolutions Canada Inc., 155 Innovation Drive, Winnipeg, MB R3T 5Y3, Canada; b The Institute for Global Risk Research, LLC, 9119 Kirkdale Road, Suite 200, Bethesda, MD 20817, USA; c Adaptive Phage Therapeutics, 708 Quince Drive, Gaithersburg, MD 20878, USA; d Emergent BioSolutions Inc., 400 Professional Drive, Gaithersburg, MD 20879, USA

**Keywords:** BioThrax®, Vaccine, Anthrax, Post-exposure prophylaxis, Drug-vaccine interaction, ciprofloxacin

## Abstract

The recommended treatment for post-exposure prophylaxis (PEP) following known/suspected exposure to *Bacillus anthracis* involves immunization with anthrax vaccine adsorbed (AVA, i.e., BioThrax® vaccine) and a course of antimicrobial therapy. A drug-vaccine interaction clinical trial was conducted to determine whether this combined treatment might modify antimicrobial exposure or vaccine immunogenicity.

A Phase 2, randomized, open-label, multi-center trial involving 154 healthy adult participants was completed to evaluate the effect of AVA immunization (three doses administered subcutaneously (SC) at weeks 0, 2 and 4) on the pharmacokinetics (PK) of ciprofloxacin, as well as the effect of ciprofloxacin administration (500 mg po bid) on the immunogenicity of AVA. PK parameters were derived using noncompartmental analysis of ciprofloxacin serum concentrations. Immunogenicity was assessed using a toxin neutralizing antibody (TNA) assay resulting in 50 % neutralization factor (NF_50_) values. Safety was assessed via reports of adverse events (AEs), clinically significant changes in laboratory parameters and vital signs, and collection of solicited local and systemic reactogenicity reactions.

Statistical analyses of the steady state (SS) and single dose PK parameters C_max_ and AUC_0-–12h_ indicated that the AVA PEP regimen did not significantly modify ciprofloxacin exposure. Comparison of the geometric mean TNA NF_50_ values between participants receiving AVA + ciprofloxacin and those receiving AVA alone showed that the combined treatment was non-inferior to AVA alone. The trial met all prospectively defined success criteria for the primary PK endpoint and for the secondary PK and immunogenicity endpoints. There were no deaths, SAEs or AEs leading to drug discontinuation or study withdrawal during the trial.

Overall, concomitant administration of ciprofloxacin and AVA produced no significant changes in the PK profile of ciprofloxacin nor in the immunogenicity of AVA. Furthermore, this trial demonstrated that the co-administration of ciprofloxacin and AVA was well tolerated in healthy adult participants.

## Highlights

• AVA + ciprofloxacin demonstrated an acceptable safety/reactogenicity profile.
• AVA + ciprofloxacin had no significant change on the immunogenicity of AVA.
• AVA regimen had no impact on the single dose of ciprofloxacin.
• AVA regimen had no impact on steady-state PK of ciprofloxacin.
• PK parameters of ciprofloxacin are consistent with literature and ciprofloxacin PI.

## Introduction

*Bacillus anthracis* (*B. anthracis*), the causative agent for anthrax disease, is considered by the US Centers for Disease Control and Prevention (CDC) to be one of the most likely biological agents to be used in a future bioterrorist attack [1]. In 2001, the release of *B. anthracis* spores in the US mail resulted in 5 deaths among the 11 patients (45 %) who contracted inhalational anthrax [2]. An estimated 32,000 people across four states and the District of Columbia were offered 60 days of antimicrobials for post-exposure prophylaxis (PEP), however adherence was poor, ranging from 21 to 64 % completing the 60-day period [3], [4]. At the time of those events, Anthrax Vaccine Adsorbed (AVA, i.e., BioThrax® vaccine), was only licensed for pre-exposure prophylaxis (PrEP), although it had been recommended by the CDC Advisory Committee on Immunization Practices (ACIP) to be part of the PEP regimen [5]. The vaccine was offered as part of a CDC Investigational New Drug (IND) protocol to those who had completed or were completing an initial 60-day course of antibiotics [6]. Participants were offered an additional 40 days of antibiotic therapy with or without three doses of AVA (to be administered at 0, 2 and 4 weeks). Additional animal studies and clinical trials were initiated to address PEP use of a three-dose series of AVA (0, 2 and 4 weeks). Study in rabbits challenged with aerosolized *B. anthracis* spores suggested that there was a survival advantage (i.e. protective threshold) when AVA was used in combination with antibiotics [7]. In two clinical studies (EBS.AVA.005, EBS.AVA.006 (NCT01491607), [8] with 350 healthy participants, the PEP regimen was well tolerated and achieved 100 % seroconversion by Day 42. Furthermore, in study EBS.AVA.006, 52.7 % of participants maintained TNA NF_50_ levels from 63 to 100 days post-vaccination. These levels were all above the predefined protective threshold as accepted by the FDA as a predictive indicator of survival in humans. To examine the potential for an interaction between AVA and recommended antibiotics for PEP, a drug-vaccine interaction clinical trial (EBS.AVA.009, NCT01753115) was conducted in healthy adult participants. This study included two treatments, where AVA was administered alone and in combination with ciprofloxacin. The twice daily regimen for ciprofloxacin was provided to participants concurrently with AVA vaccine to allow for comparison of ciprofloxacin pharmacokinetic parameters with respect to the test (Day 44 following three doses of AVA received on Day 5, 20 and 34) and reference (prior to AVA vaccination on Day 5) treatments. The primary objective of the trial was to assess steady-state pharmacokinetic (PK) parameters including area under the concentration curve from zero to 12 h (AUC_0-12hr_) and maximum serum concentration (C_max_) of ciprofloxacin when orally administered prior to and following subcutaneous (SC) administration of a three-dose series of AVA. Secondary objectives were: (1) to evaluate non-inferiority of the immune response when immunization with AVA took place concomitantly with ciprofloxacin dosing compared to AVA immunization alone, and (2) to evaluate the safety of AVA and ciprofloxacin administrations.

## Methods

### Investigational products and administration procedures

AVA is prepared from cell-free culture filtrates of an avirulent, non-encapsulated strain of *B. anthracis*. The final product contains culture fluid proteins including the *B. anthracis* protective antigen (PA), 1.2 mg/mL aluminum as an adjuvant; and 25 mcg/mL benzethonium chloride and 100 mcg/mL formaldehyde as preservatives [9]. AVA was administered SC as a three-0.5 mL dose series with each subsequent dose administered two weeks apart using alternating arms for each injection. The AVA used for this trial was supplied in 5 mL multi-dose vials (10 doses/vial) stored at 2–8 °C. Commercial lot FAV392A was used for the study.

Ciprofloxacin hydrochloride was provided as film-coated tablets at a strength of 500 mg ciprofloxacin equivalent. The tablets were manufactured by TEVA Pharmaceuticals. The sites converted the cartons into labeled kits and distributed them to participants in Arms 1 and 2 (one kit per ciprofloxacin course). Two lots of ciprofloxacin were used in this trial; lot B120508 and lot B130335.

### Study design

The trial (NCT01753115) was a randomized, open-label, phase 2, three-center study in the USA to investigate the potential interactions of ciprofloxacin and AVA in adult healthy participants, 18 to 45 years of age, inclusive (Table 1). The study was conducted in accordance with the Declaration of Helsinki and Good Clinical Practice. After obtaining the informed consent form (ICF), 237 participants were screened with 154 meeting the eligibility criteria. A central randomization process, with stratification by gender and age (18 to ≤ 30 vs. 31–45 years) was then used to assign these participants (N = 154) at a ratio of 1:1:2 to one of three arms: Arm 1 = AVA + ciprofloxacin with ciprofloxacin PK component (N = 44), Arm 2 = AVA + ciprofloxacin without ciprofloxacin PK component (N = 40), and Arm 3 = AVA alone (N = 70). To support the primary objective of the study, Arms 1 and 2 were to include a minimum of 20 participants to ensure 80 % power in assessing an interaction between AVA and ciprofloxacin. Enrollment of 44 and 40 participants into Arm 1 and Arm 2 respectively, provided sufficient group sizes, once dropouts were taken into account, that all participants who had completed both pre- and post- vaccination could be included in the analysis.

**Table 1:** Demographic and Baseline Characteristic Information for Per Protocol, AVA Immunogenicity and Intent to Treat Populations.

Characteristic		Analysis Populations			
		Per Protocol (PP)	AVA Immunogenicity		Intent to Treat (ITT)
			Test Treatment	Reference Treatment	
		Arm 1^a^	Arm 1 + 2^b^	Arm 3^c^	All Participants
		N = 32	N = 50	N = 50	N = 154^d^
Gender [n (%)]	Male	19 (59.4)	26 (52.0)	26 (52.0)	78 (50.6)
	Female	13 (40.6)	24 (48.0)	24 (48.0)	76 (49.4)
Race [n (%)]	White	19 (59.4)	30 (60.0)	27 (54.0)	79 (51.3)
	Black or African American	13 (40.6)	19(38.0)	21 (42.0)	68 (44.2)
	Asian	0	0	1 (2.0)	3 (1.9)
	American Indian or Alaskan Native	0	1 (2.0)	1 (2.0)	3 (1.9)
	Multiple	0	0	0	1 (0.6)
Ethnicity [n (%)]	Hispanic or Latino	5 (15.6)	8 (16.0)	5 (10.0)	19 (12.3)
	Not Hispanic or Latino	27 (84.4)	42 (84.0)	45 (90.0)	135 (87.7)
Age (years)	Mean (SD)	30.8 (8.91)	31.4 (8.79)	30.6 (7.35)	30.4 (7.88)
	Min, Max	18, 45	18, 45	19, 44	18, 45
Age group [n (%)]	18–30 Years	15 (46.9)	23 (46.0)	24 (48.0)	77 (50.0)
	>30 Years	17 (53.1)	27 (54.0)	26 (52.0)	77 (50.0)
Height (cm)	Mean (SD)	173.7 (10.51)	172.8 (9.53)	170.8 (8.94)	171.5 (10.27)
	Min, Max	157.4, 200.6	157.4, 200.6	148.0, 186.0	148.0, 205.0
Weight (kg)	Mean (SD)	94.8 (26.32)	92.2 (28.73)	83.9 (27.71)	87.9 (26.36)
	Min, Max	62.0, 165.0	53.0, 196.0	50.0, 207.0	50.0, 207.0

AVA = anthrax vaccine adsorbed; ITT = intent-to-treat; n (%) = percentage of subjects in that treatment or population; PK = pharmacokinetics; PP = per protocol; SD = standard deviation

a Arm 1 = AVA + ciprofloxacin with ciprofloxacin PK.

b Arm 2 = AVA + ciprofloxacin without ciprofloxacin PK.

c Arm 3 = AVA.

d The ITT population included the following subjects: Arm 1 = 44 subjects, Arm 2 = 40 subjects and Arm 3 = 70 subjects. The difference in the subject counts for the PP and AVA immunogenicity populations, relative to the ITT population, are detailed in Fig. 2.

**Fig. 1:**
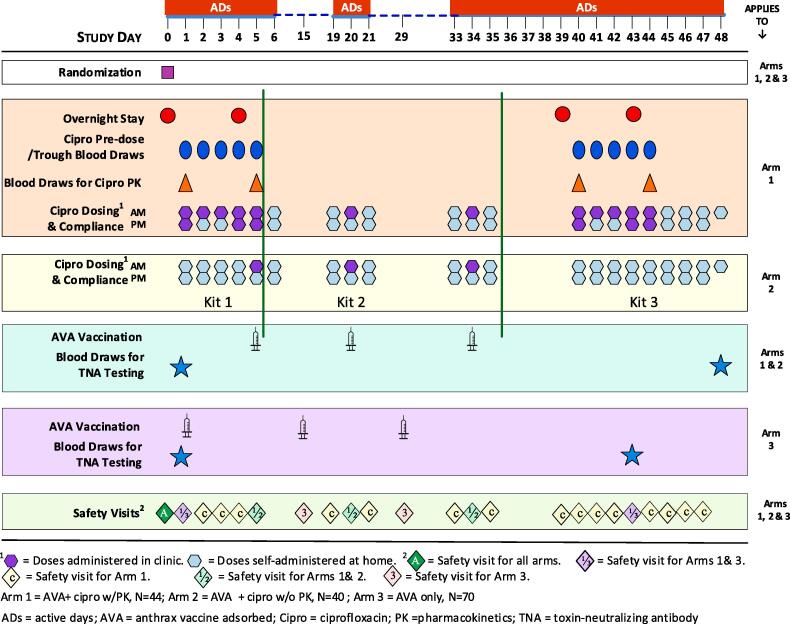
Study Design Schematic.

A study schematic for the trial is shown in Fig. 1. Only active days are shown, i.e., days when participants either came to the clinic for blood draws, vaccination or took ciprofloxacin dose(s) at home. Ciprofloxacin was administered at 500 mg po bid until morning of the final study visit and AVA (0.5 mL) was administered by SC injection, 3 doses two weeks apart.

This study was completed at three sites in the USA as follows: Site 1 = Research Across America, 9 Medical Parkway, Plaza 4, Suite 202, Dallas, TX 75234; Site 2 = The Center for Pharmaceutical Research, 1010 Carondelet Drive, Suite 426, Kansas City, MO 64114, Site 3 = Meridian Clinical Research, 3319 N 107th Street, Omaha, NE 68134.

### Analysis of pharmacokinetics

Ciprofloxacin concentrations were determined in human serum samples using deuterium-labelled ciprofloxacin as an internal standard at ICON Development Solutions in Whitesboro, New York. Analyses were performed using a validated liquid chromatography with tandem mass spectrometry (LC-MS/MS) method involving a Shimadzu liquid chromatography system with a PE Sciex mass spectrometer with Ionics HSID Interface. The lower limit of quantitation for ciprofloxacin was established at 50.0 ng/mL.

Blood samples for the determination of ciprofloxacin PK were collected only from Arm 1 participants to determine pre- and post-vaccination trough levels. The samples on Days 1 through Day 5 were used to determine the single dose and steady-state PK of ciprofloxacin, respectively, prior to vaccination. Samples collected on Day 40 through Day 44 were used to evaluate the potential impact of the complete AVA three-dose PEP vaccination regimen on the single dose and steady-state PK of ciprofloxacin, respectively.

### Immunogenicity assessment

Blood samples for TNA assessments in Arms 1, 2 and 3 were collected on Day 1 (baseline) and then 14 days after the last vaccination for the AVA + ciprofloxacin and AVA alone treatment groups. TNA titers at each study visit were summarized in terms of geometric mean titers (GMTs) and their corresponding 95 % confidence intervals (CIs). GMTs and their 95 % CIs were calculated using normal approximation assuming neutralizing antibody titers were log-normally distributed. The CIs for GMTs were computed by taking the anti-logarithms of the lower and upper limits of the CIs for log10 GMT. Samples were tested by Battelle Memorial Institute (West Jefferson, Ohio, United States) using a validated, high-throughput, cell-based bioassay that measured neutralization of anthrax lethal toxin (LT) by antibodies generated in response to vaccination with AVA. Values for NF_50_ (50 % neutralization factor) were calculated as the ratio of 50 % effective dilution (ED_50_) of the test sample to ED_50_ of a reference serum identified as AVR801.

### Safety assessments

Medical history was obtained at screening by direct interview, which included prior immunization history. Safety in all three arms was assessed by physical examinations (PEs), vital signs, laboratory (hematology, serum chemistry, urinalysis), treatment emergent adverse events (TEAE) assessments, collection of concomitant medication information, and solicited injection site and systemic reactogenicity reactions. Solicited injection site reactions consisted of tenderness, pain, lump, warmth, swelling, arm motion limitation (AML), redness, itching and bruise, while solicited systemic reactions consisted of muscle ache, fatigue/tiredness, headache, and fever. Reactogenicity reactions were assessed and reported by participants using e-diaries for seven days after each vaccination, or longer if reactions persisted beyond seven days. Reactogenicity also was evaluated by the investigator in the clinic prior to administering the next dose of study vaccine and at the final study in-clinic visit (14 days after last vaccination). Grade 3 and above reactogenicity reactions were evaluated and recorded as TEAEs.

The TEAEs were assessed at every (scheduled and unscheduled) clinic visit for Arms 1, 2, and 3. Any participant who discontinued treatment was encouraged to continue safety follow-up by attending subsequent visits through Day 43 (Arm 3) or through Day 48 (Arms 1 and 2).

### Statistical methods and study populations

A statistical analysis plan (SAP) for the analysis of PK parameters, immunogenicity and safety data was prepared before database lock. The intent-to treat (ITT) population was defined as all participants who were randomized; this population was used for all demographic analyses. The safety population was defined as all randomized participants who received at least one dose of either ciprofloxacin or AVA; this population was used for all safety analyses. The per protocol (PP) population was defined as all participants in Arm 1 who received any dose of ciprofloxacin, had adequate PK data on Day 5 and Day 44, had received all three AVA doses within the study-specified windows, and who had no key protocol deviations (e.g., insufficient blood sample) that would be expected to affect the ciprofloxacin PK assessment. This population was used for PK analyses. The AVA immunogenicity population was defined as all participants who met the following criteria: (1) received the correct dose (0.5 mL) of three AVA vaccinations within the study-specified windows, (2) had immunogenicity samples taken within the study-specified time windows and had a valid result for immunogenicity two weeks following the last vaccination, (3) had no evidence of previous anthrax vaccination, and (4) received AVA vaccine that was maintained at the proper temperature. The AVA immunogenicity population was used for the immunogenicity analyses. Baseline was defined as the last available value before any dosing (either ciprofloxacin or AVA). Using the TNA NF_50_ values two weeks after the last vaccination as the endpoint, Arms 1 + 2 (AVA + ciprofloxacin) values were compared with the Arm 3 (AVA alone) values. If the lower bound of the 95 % CIs for the geometric mean ratio (GMT_Arms 1+2_/GMT_Arm 3_) was greater than 0.67, AVA + ciprofloxacin co-administration would be deemed non-inferior to AVA alone.

The ciprofloxacin PK parameters were calculated from the ciprofloxacin concentration–time data using noncompartmental analysis methods with Phoenix WinNonlin™. Actual sampling times were used for the analysis.

For Arm 1, assessment of interaction was made by evaluating the geometric mean of within-subject ratios (GMRs) for C_max_ and AUC_0-12h_ for ciprofloxacin following 3 doses of AVA on Day 44 (test) compared to those determined prior to AVA vaccination on Day 5 (reference). If the 90 % CIs for both GMRs fell completely within the equivalence bounds of 0.80 and 1.25, then it is concluded that AVA vaccination had no effect on the steady-state or single dose PK of ciprofloxacin.

The trough levels of ciprofloxacin were also evaluated by comparing Days 1, 2, 3, 4, and 5 and Days 40, 41, 42, 43, and 44 to evaluate time to steady-state before and after AVA vaccination, using the NOSTASOT (No-Statistical-Significance-Of-Trend) methodology[10].

All safety analyses were carried out using the safety population. Safety parameters are presented by arm and treatment, with continuous variables being summarized using mean, standard deviation (SD), median, minimum, and maximum. Note that TEAEs were collected from Day 1 through Day 48 for Arms 1 + 2 and from Day 1 through Day 43 for Arm 3.

## Results

### Baseline characteristics and disposition of participants

**Fig. 2:**
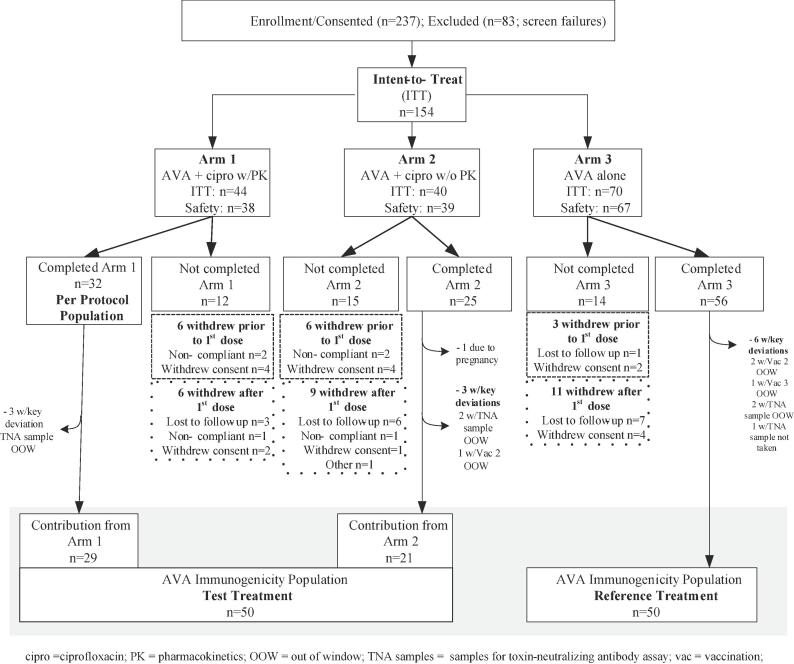
Participant Disposition.

Disposition of participants by analysis population is presented in Fig. 2. Out of 237 participants who signed the ICF and were screened for participation in the study, 154 met the entry criteria and were randomized into one of the three study arms becoming the ITT population. The enrollment, treatment and follow-up of participants occurred from December 14th, 2012, to the last subject last visit (LSLV) on August 4th, 2013.

Out of 83 participants who were screened but not randomized, 66 did not meet one or more eligibility criteria, 11 because their strata closed, 3 because the study closed before they could be randomized, 2 withdrew consent, and 1 was a screen failure because the site had insufficient ciprofloxacin for enrollment.

Twelve participants were excluded from the PP population due to non-compliance, withdrawal of consent or being lost to follow up; none were excluded due to protocol deviations.

There were 54 excluded from the AVA immunogenicity population. Forty-one (41) of these participants were withdrawn from the study due to non-compliance (N = 6), exclusionary drug use (N = 1), withdrawal of consent (N = 17) or being lost to follow up (N = 17); one was discontinued due to pregnancy, prior to receiving second AVA vaccination and the remaining 12 were excluded due to protocol deviations such as vaccinations or TNA samples taken out of the time window or missed. The AVA immunogenicity population included 100 participants, 50 in the AVA + ciprofloxacin and 50 in the AVA alone treatment arm.

Ten participants were discontinued from the study following randomization but prior to receiving any study treatment and were excluded from the safety population: six participants in Arm 1; one participant in Arm 2 and three participants in Arm 3. Six withdrew consent, three were discontinued for non-compliance and one was lost to follow-up.

Participant enrollment amongst the three sites was distributed evenly with Sites 1, 2 and 3 enrolling 35.1 % (54/154), 31.2 % (48/154); and 33.8 % (52/154) of participants in the ITT population, respectively. By treatment, Sites 1, 2, and 3 enrolled 36.9 % (31/84); 29.8 % (25/84); and 33.3 % (28/84) of the participants who received AVA + ciprofloxacin (Arms 1 + 2), respectively, compared to 32.9 % (23/70); 32.9 % (23/70); and 34.3 % (24/70) of the participants who received AVA alone (Arm 3).

### Pharmacokinetics evaluation

The primary objective was to evaluate the equivalence of the geometric mean steady-state AUC_0–12 hr_ and C_max_ values for ciprofloxacin before and after vaccination with the full PEP AVA (3-dose) regimen. The pharmacokinetic parameters used to determine the possible impact of vaccination on ciprofloxacin PK are shown in Table 2.

**Table 2:** Summary of Ciprofloxacin Pharmacokinetic Parameters by Day.

**Parameter (units)**	**Day 1**	**Day 5**	**Day 40**	**Day 44**
AUC_0-12h_ (ng*hr/mL)	N = 317888.0 (29.04)	N = 3211569.6 (26.38)	N = 32 8641.7 (40.23)	N = 32 10352.0 (29.15)
AUC_0-inf_ (ng*hr/mL)	N = 31 9636.7 (32.29)	N = 32 14966.2 (27.29)	N = 32 10790.6 (39.54)	N = 32 13230.4 (30.84)
C_max_ (ng/mL)	N = 32 2004.0 (34.16)	N = 32 2621.7 (25.65)	N = 32 2120.3 (38.78)	N = 32 2465.4 (25.23)
T_max_ (hr)	N = 32 1.225 (0.50–5.00)	N = 32 1.025 (0.27–4.00)	N = 32 1.00 (0.48–4.03)	N = 32 0.760 (0.47–4.00)
T_1/2_ (hr)	N = 31 4.933 (22.82)	N = 32 5.969 (36.60)	N = 32 5.653 (41.00)	N = 32 5.926 (40.08)

AUC_0-12h_ = area under curve at time 0–12 h; AUC_0-inf_ = area under curve at time 0 to infinity; C_max_ = maximum concentration; N = number of participants; T_1/2 =_ half-life; T_max_ = time to maximum concentration

Note: Summaries are presented as arithmetic mean (%CV) except for T_max_ which is summarized as median (min-max).

Day 1 = parameters calculated from ciprofloxacin concentrations on Day 1, prior to AVA vaccination on Day 1.

Day 5 = parameters calculated from ciprofloxacin concentrations on Day 5, prior to AVA vaccination on Day 5.

Day 40 = parameters calculated from ciprofloxacin concentrations on Day 40, following AVA vaccination 3 on Day 34.

Day 44 = parameters calculated from ciprofloxacin concentrations on Day 44, following AVA vaccination 3 on Day 34.

To evaluate the possible impact of vaccination on the PK parameters of ciprofloxacin, the C_max_ and AUC_0-12h_ GMRs for Day 44 over Day 5 (ciprofloxacin at steady state, before [Day 5] and after [Day44], i.e., over the course of the complete PEP AVA vaccination regimen) were calculated with their 90 % CIs. The resulting 90 % CIs, 0.83 to 0.95 for AUC_0-12h_, and, 0.85 to 1.05 for C_max_, fell within the 0.80 to 1.25 limits for demonstrating equivalence and indicate that three doses of AVA did not have an impact on ciprofloxacin exposure at steady state. Analysis for Day 1 vs. Day 40 (ciprofloxacin single dose after the before [Day 1] and after [Day 40] the complete PEP AVA regimen) was carried out in a similar fashion. The resulting 90 % CIs of GMRs, 0.99 to 1.19 for AUC_0-12h_, and, 0.91 to 1.22 for C_max_, fell within the 0.80 to 1.25 limits for demonstrating equivalence and indicated that three doses of AVA did not have an impact on the single dose ciprofloxacin exposure.

### Analysis of immunogenicity

Evaluating the non-inferiority of the immune response when the full regimen of AVA was administered in combination with ciprofloxacin to AVA alone was a secondary objective of the study. Using the TNA NF_50_ values two weeks after the last vaccination as the endpoint, Arms 1 + 2 (AVA + ciprofloxacin) values were compared with the Arm 3 (AVA alone) values. If the lower bound of the 95 % CIs for the geometric mean ratio (GMT_Arms 1+2_/GMT_Arm 3_) was greater than 0.67, AVA + ciprofloxacin co-administration would be deemed non-inferior to AVA alone. A total of 54 participants (15 from Arm 1, 19 from Arm 2 and 20 from Arm 3) were excluded from this analysis leaving an AVA immunogenicity population of 100 subjects, which was appropriately powered for the assessment of non-inferiority. The point estimate for the ratio was 1.27 and the 95 % CIs were (0.90, 1.79). Since the lower CI was > 0.67, AVA + ciprofloxacin co-administration was non-inferior to AVA alone (Table 3).

**Table 3:** Non-Inferiority Analysis of TNA NF_50_ GMTs between Test and Reference Treatments Two Weeks Post-Vaccination.

**GMT NF_50_**					
**Test Treatment (N = 50)** **(Arms 1 + 2)^ab^**		**Reference Treatment (N = 50)** **(Arm 3)^c^**		**Ratio of GMTs** **(Arms 1 + 2/Arm 3)**	**95 % CI of Ratio**
**GMT**	**95 % CI**	**GMT**	**95 % CI**		
1.17	0.91, 1.50	0.92	0.72, 1.78	1.27	0.90^d^, 1.79

AVA = anthrax vaccine adsorbed; CI = confidence interval; GMT = geometric mean titers; N = number of participants; NF_50_ = 50 % neutralizing factor.

^a^Arm 1 = AVA + ciprofloxacin with ciprofloxacin PK.

^b^Arm 2 = AVA + ciprofloxacin without ciprofloxacin PK.

^c^ Arm 3 = AVA.

^d^ This meets the noninferiority success criteria of the lower bound 95 % CI being > 0.67.

The immunogenicity NF_50_ values were summarized by gender, age (18 to ≤ 30 years and > 30 years), race (White, non-White), ethnicity (Hispanic, Non-Hispanic), and study site. The demographic subgroup outcomes were similar to the immunogenicity results from previous AVA studies {Wright *et al.*, 2013; Ionin *et al*., 2013; Emergent 2007} with slightly higher TNA NF_50_ GMT values in females (1.14) vs. males (0.96), participants of age 18–30 years (1.12) vs. participants > 30 years (0.91), and White/Caucasian participants (1.21) vs. non-Caucasian participants (0.85).

### Safety

There were no deaths, serious adverse events (SAEs) or TEAEs leading to discontinuation of treatment or withdrawal from the study. Among the safety population of 144 participants, 47 (32.6 %) had TEAEs. Eleven of the 144 (7.6 %) had TEAEs assessed as severe, 24 of 144 (16.7 %) had TEAEs assessed as moderate and 30 of 144 (20.8 %) had TEAEs assessed as mild. Of the 11 participants in Arms 1 + 2 with severe TEAEs, there were eight participants who experienced nine TEAEs; three of which were solicited injection site reactions and one headache.

Shown in Table 4 are the TEAEs by MedDRA preferred term (PT) that occurred in at least 2 % of participants in the safety population. The most common TEAEs in the safety population were headache (7.6 %), nausea (4.9 %) and upper respiratory infection (4.2 %). Of those TEAEs that occurred with an incidence of greater than 5 % in participants in any arm, there were only three statistically significant reactogenicity TEAEs that were more frequent in AVA alone vs. AVA + ciprofloxacin: myalgia (7.5 %), injection site mass (6.0 %) and injection site pain (6.0 %).

**Table 4:** Treatment-Emergent Adverse Events by Preferred Term Occurring in at Least 2% of Participants– Safety Population.

MedDRA Preferred Term	Treatment		
	Arm 1 + 2^a^	Arm 3^b^	All
	N = 77	N = 67	N = 144
	n (%)	n (%)	n (%)
At Least One TEAE^c^, ^d^	26 (33.8)	21 (31.3)	47 (32.6)
§Headache	7 (9.1)	4 (6.0)	11 (7.6)
Nausea	2 (2.6)	5 (7.5)	7 (4.9)
Upper respiratory tract infection	3 (3.9)	3 (4.5)	6 (4.2)
§Fatigue	2 (2.6)	3 (4.5)	5 (3.5)
§Myalgia	0	5 (7.5)	5 (3.5)
†Injection site erythema	2 (2.6)	2 (3.0)	4 (2.8)
†Injection site mass	0	4 (6.0)	4 (2.8)
†Injection site pain	0	4 (6.0)	4 (2.8)
§Arthralgia	2 (2.6)	1 (1.5)	3 (2.1)
Vomiting	1 (1.3)	2 (3.0)	3 (2.1)
†Joint range of motion decreased^e^	0	2 (3.0)	2 (1.4)
Vulvovaginal pruritus	2 (2.6)	0	2 (1.4)
Cough	2 (2.6)	0	2 (1.4)

N = number of participants randomized in the safety population; n = number of participants with adverse events within each group exposed to the treatment; TEAE = treatment emergent adverse events.

Arm 1 = AVA + ciprofloxacin with ciprofloxacin PK.

Arm 2 = AVA + ciprofloxacin without ciprofloxacin PK.

Arm 3 = AVA.

a TEAEs assessed from Day 0 to Day 48.

b TEAEs assessed from Day 0 to Day 43.

c A TEAE was any unsolicited adverse event that either was not present prior to treatment and appeared following treatment or which increased in severity or frequency following treatment. Participants with adverse events in more than one category were counted once in each of those categories. Severe (Grade 3) solicited injection site and solicited systemic reactions recorded in participants’ e-diaries were also to be recorded as TEAEs that were MedDRA coded and included in TEAE tables; however, the principal investigator was not required to maintain the same severity grade.

d TEAEs marked with § and †were also solicited systemic or solicited injection site reactions, respectively, which participants recorded in their e-diaries and which could be converted to MedDRA TEAEs (and be included in TEAE tables) if reported as severe reactions in participant e-diaries.

e This is the MedDRA preferred term used for arm motion limitation (AML).

**Fig. 3:**
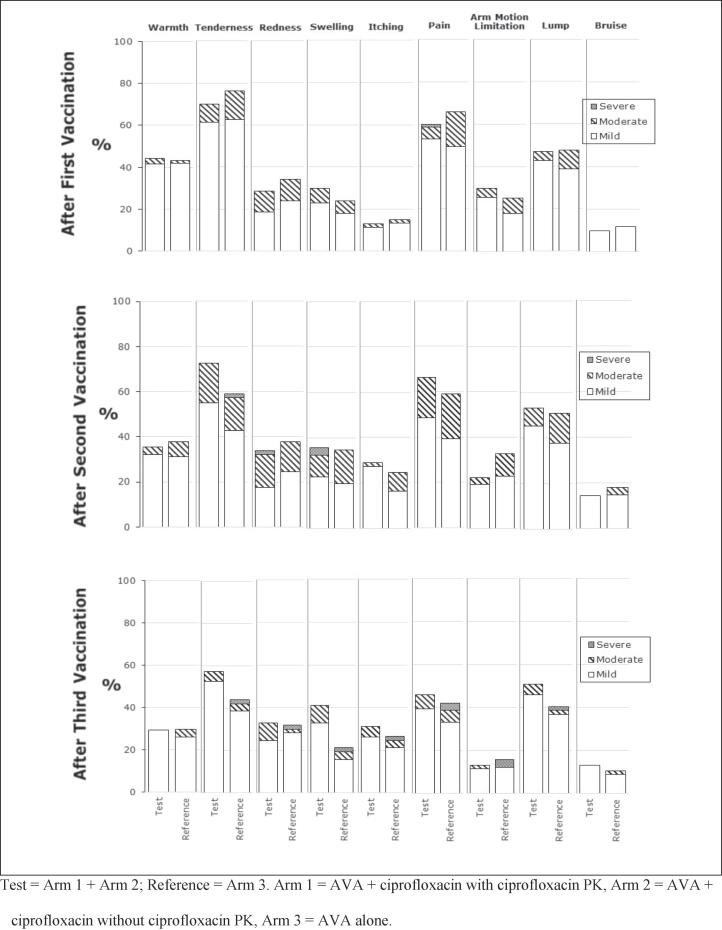
Solicited injection site reactogenicity responses after each vaccination (e-diary data).

Participants were required to fill out a daily e-diary. The e-diary collected a variety of information throughout the study, including oral temperature, times when participants in Arms 1 and 2 self-administered ciprofloxacin, changes in health status throughout the study and use of concomitant medication. Additionally, injection site reactogenicity responses were also captured following each vaccination (Fig. 3).

**Fig. 4:**
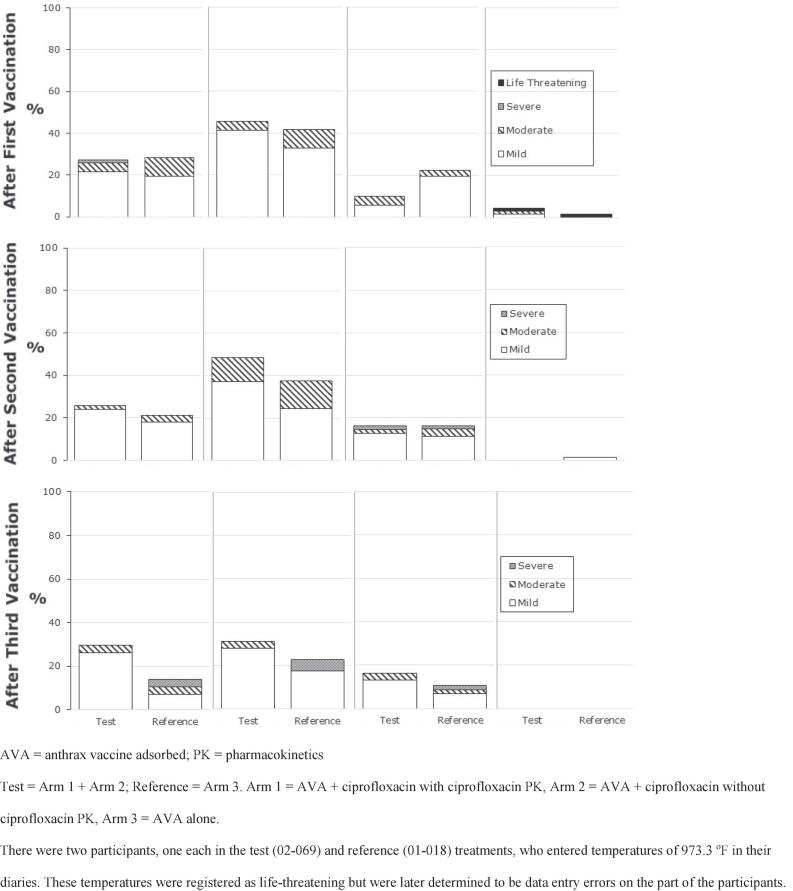
Solicited systemic reactogenicity responses after each vaccination (e-diary data).

After the first, second and third vaccinations, 83.1 %, 83.3 %, and 69.0 % of participants who received AVA + ciprofloxacin, respectively, reported any injection site reactions in their e-diaries, while the comparable percentages for those who received AVA alone were 88.5 %, 78.9 %, and 56.9 %. Summary information for e-diary solicited systemic reaction data following each vaccination is provided in Fig. 4.

After the first, second and third vaccinations, 52.9 %, 54.8 %, and 40.0 % of the participants who received AVA + ciprofloxacin, respectively, reported at least one systemic reaction in e-diaries, while the comparable percentages for those who received AVA alone were 50.0 %, 43.1 %, and 29.6 %, respectively. The rank order of incidence after all three vaccination was the same for both the test and reference treatments and was muscle ache > fatigue/tiredness > headache > fever.

Most of the systemic reactions recorded by participants in all arms after any vaccination were mild in severity. Three participants who received AVA alone reported severe systemic reactions following the third vaccination compared to no participants who received AVA + ciprofloxacin. Overall, the incidence of systemic reactions was similar between participants who received AVA + ciprofloxacin and those who received AVA alone.

## Discussion

The primary objective of this trial was to evaluate whether a three-dose series of AVA administered at 0, 2, and 4 weeks (i.e.*,* two weeks apart) affected exposure to ciprofloxacin (i.e.*,* modified its PK profile) when the antibiotic was administered concomitantly using the recommended dosing regimen for anthrax (i.e., 500 mg bid). A secondary objective of this study was to assess whether the immune response (i.e., TNA NF_50_ values) following three doses of AVA was non-inferior to AVA co-administered with ciprofloxacin. This study also monitored safety of the concurrent administration of ciprofloxacin + AVA and AVA alone.

An analysis was performed to evaluate the impact of AVA administration on the PK of ciprofloxacin for Day 44 vs. Day 5. The 90 % CIs for the geometric mean of the within-subject ratio (AVA + ciprofloxacin:AVA alone) for Day 44 vs. Day 5 were 0.83 to 0.95 for AUC_0-12h,_ and 0.85 to 1.05 for C_max_. Since both CIs were entirely contained within the pre-specified 0.80 to 1.25 limits, the success criterion for the primary endpoints was met. These findings indicate that AVA did not affect the steady-state PK of ciprofloxacin. Furthermore, a comparison of AUC_0-12h_ and C_max_ on Day 40 (ciprofloxacin single dose, after AVA administration) vs. Day 1 (ciprofloxacin single dose, before AVA administration) using the same success criterion did not identify an effect of AVA. Thus, AVA did not affect the single dose PK of ciprofloxacin and the secondary PK endpoint criteria was met. This study supports the approved PEP use of AVA in conjunction with antibacterial drugs.

In addition, the predefined success criterion was met for the secondary immunogenicity endpoint analysis. Using the TNA (NF_50_) GMT ratio of Arms 1 + 2 (AVA + ciprofloxacin) to Arm 3 (AVA alone), the lower bound of the 95 % CI was 0.90, i.e., greater than the pre-specified non-inferiority limit of 0.67. Thus, the test treatment can be deemed non-inferior to the reference treatment (AVA).

AVA and ciprofloxacin when co-administered had an acceptable safety and reactogenicity profile, equivalent to AVA when administered alone. There were no reports of deaths, SAEs nor TEAEs leading to drug discontinuation or study withdrawal. Furthermore, there were no remarkable differences in the incidence or severity of TEAEs or solicited injection or systemic reactions between participants who received AVA and ciprofloxacin concomitantly vs. those who received AVA alone. In addition, the incidence of local (injection site) and systemic adverse reactions were comparable to those reported in the prescribing information for AVA. Specifically, injection site tenderness, pain, lump, warmth, redness (erythema), swelling (edema), and arm motion limitation were the most common TEAEs reported in this study and are in-line with historical safety data. The most common systemic TEAEs reported were muscle aches, fatigue, and headache, which were comparable to historical safety data. The safety signals reported in this study support the BioThrax® vaccine prescribing information and provide further confidence in the safety of this vaccine for PEP administration.

In 2000, AVA vaccination was recommended by ACIP for PEP treatment of exposure to *B. anthracis* [5]*.* Following the 2001 release of *B. anthracis* spores in the US mail, thousands of people across 6 states were provided 60 days of antibiotics for PEP [3], [4]. As part of the emergency public health response to the first documented bioterrorism-associated anthrax attack, the CDC filed an IND in Oct 2001 to evaluate antibiotic treatment (doxycycline, ciprofloxacin or amoxicillin) with or without 3 doses of AVA out to 100 days post exposure. Following IND initiation in December 2001 to program completion in September 2002, 1727 people were enrolled, of which 1528 (88 %) opted to received only the 40-day antibiotic therapy and 199 (12 %) opted to receive the 40-day supply of antibiotic therapy plus 3 doses of AVA [6]. The use of antibiotics postexposure can reduce the progression of anthrax disease but does not protect against germination of spores that may remain in the body after antibiotic treatment. To further support the use of AVA with antibiotics, a study in rabbits was completed with a *B. anthracis* challenge followed by treatment with levofloxacin with or without AVA. This study demonstrated a significant increase in survival rates amongst vaccinated animals compared to those treated with antibiotic alone [7], and supported a protective antibody threshold that was confirmed in a subsequent safety and immunogenicity bridging trial in humans [8]. The current drug-vaccine interaction study has demonstrated that ciprofloxacin and AVA can be safely co-administered as part of a PEP regimen, without impacting either ciprofloxacin PK or the antibody response to AVA.

## Conclusions

When ciprofloxacin and AVA were concomitantly administered, there was no effect on either ciprofloxacin PK or AVA immunogenicity. This study met the prospectively defined success criteria for the primary PK and secondary immunogenicity analyses. AVA vaccine administered as three SC doses at 0, 2 and 4 weeks, when given in combination with daily oral ciprofloxacin doses of 1000 mg or without ciprofloxacin, demonstrated a well-tolerated safety/reactogenicity profile. This study provides further confidence in the use of this vaccine for PEP administration and was supportive of the approval of BioThrax® vaccine for postexposure prophylaxis in 2015.

## Funding

This project has been funded in part with federal funds from the Biomedical Advanced Research and Development Authority, Department of Health and Human services, under Contract No. HHSO100200700037C. The funding organization had no role in the design and conduct of the study; collection, management, analysis, and interpretation of the data, preparation, review or approval of the manuscript; and decision to submit the manuscript for publication.

## CRediT authorship contribution statement

**David Cassie:** . **Janice Longstreth:** Writing – review & editing. **Robert Hopkins:** Writing – original draft, Writing – review & editing. **Ericka Hunter-Stitt:** Conceptualization, Formal analysis, Investigation, Methodology, Project administration, Writing – review & editing. **Bojan Drobic:** . **Melisa Bellani:** Writing – original draft, Writing – review & editing.

## Declaration of competing interest

The authors declare that they have no known competing financial interests or personal relationships that could have appeared to influence the work reported in this paper.

## Data Availability

The data that has been used is confidential.
